# A time-series study of percutaneous closure of patent foramen ovale: premature adoption?

**DOI:** 10.1136/openhrt-2015-000313

**Published:** 2016-01-04

**Authors:** Kian Nian Lew, Gianni D Angelini, William Hollingworth

**Affiliations:** 1 Faculty of Medicine and Dentistry, University of Bristol, Bristol, UK; 2 Bristol Heart Institute, University Hospitals Bristol NHS Foundation Trust, Bristol, UK; 3 School of Social and Community Medicine, University of Bristol, Bristol, UK

**Keywords:** CONGENITAL HEART DISEASE

## Abstract

**Objectives:**

To evaluate the impact of National Institute for Health and Care Excellence (NICE) guidance in January 2005 and subsequent trial evidence on the adoption of percutaneous closure of patent foramen ovale (PCPFO).

**Methods:**

A retrospective time series study was conducted using the Inpatient Hospital Episode Statistics (HES) England. A total of 3801 patients, aged ≥18 and ≤60 years, who had PCPFO from 1 April 2006 to 31 March 2012 in England. Percentage change annualised (PCA) in PCPFO procedure rates between initial NICE guidance and publication of trial results was analysed.

**Results:**

Between Quarter 2, 2006 and Quarter 4, 2009, 2163 PCPFO procedures were performed, with an increasing PCA of 48.4%. The procedure rate peaked before the presentation of equivocal results from the first randomised controlled trial (RCT) in late 2010, and declined between Quarter 4, 2009 and Quarter 4, 2011 (PCA=−15.3%). Of more than 2300 patients recruited to three RCTs, only 71 were recruited in English hospitals.

**Conclusions:**

PCPFO was rapidly adopted after the publication of initial NICE guidance despite the absence of RCT evidence of efficacy. Very few English patients participated in international RCTs of PCPFO, suggesting that NICE recommendations also failed to encourage the generation of RCT evidence.

**Key questions**

**What is already known about this subject?**

- In January 2005, National Institute for Health and Care Excellence (NICE) guidance recommended that percutaneous closure of patent foramen ovale (PCPFO) could be used in the secondary prevention of stroke/transient ischaemic attack for audit or research purposes, despite lack of evidence from randomised controlled trials (RCTs).

**What does this study add?**

- PCPFO had been rapidly adopted with a percentage change annualised of 48.4% in the first 4 years since initial NICE guidance.
- RCTs of PCPFO have struggled to recruit, only a small number of English centres and patients contributed to RCTs.
- The increased adoption of PCPFO after initial NICE guidance despite the absence of RCT evidence suggests that NICE guidance on PCPFO encouraged premature adoption of PCPFO and may have stifled generation of RCT evidence.

**How might this impact on clinical practice?**

- Clinicians must be cautious in embracing nascent medical interventions until the safety and efficacy of the medical interventions are established.
- Greater clinicians’ participation is needed in the recruitment of patients into RCT.

## Introduction

Patent foramen ovale is postulated to increase the risk of ischaemic stroke/transient ischaemic attack (TIA) through the paradoxical embolism mechanism which accounted for about 32% of ischaemic stroke/TIA.^1^ ^2^ Percutaneous closure of patent foramen ovale (PCPFO) is a relatively new day case interventional procedure that might reduce risk of the recurrence of stroke/TIA with low complication rates. Although rare, potential periprocedural major complications of PCPFO include pericardial tamponade (0.2%; 95% CI 0.1% to 0.2%), cerebrovascular events (0.2%; 95% CI 0.0% to 0.3%), device embolism requiring surgery (0.1%; 95% CI 0.0% to 0.2%) and retroperitoneal haematoma (0.1%; 95% CI 0.0% to 0.1%). Long-term major complications include arrhythmias (3.3%; 95% CI 1.1% to 5.5%), cerebrovascular events (1.5%; 95% CI 1.0% to 2.0%) and device thrombosis (0.7%; 95% CI 0.4% to 1.0%).^3^

In 2005, despite lack of randomised controlled trial (RCT) evidence, the National Institute for Health and Care Excellence (NICE) interventional procedures guidance recommended that PCPFO could be used in the secondary prevention of stroke/TIA with audit and further research of safety and efficacy.^4^ Three RCTs^5–7^ published since 2010 comparing PCPFO with medical therapy found some evidence of reduction in the composite outcome of death or stroke/TIA, but the results were not statistically significant. Meta-analyses that combine stroke and TIA outcomes from these three RCTs find weak evidence that PCPFO is effective (eg, HR=0.59; 95% CI 0.36 to 0.97).^8^ Those that look at outcomes individually find weaker evidence (eg, HR=0.62; 95% CI 0.34 to 1.11)^9^ and tend to reach more negative conclusions.^10^ ^11^ Revised NICE guidance in 2013 recommended that PCPFO was suitable for routine National Health Service (NHS) use.^12^ Our study aims to evaluate the impact of NICE guidance in 2005 and dissemination of subsequent trial evidence from 2010 on the adoption of PCPFO.

## Methods

A time series study was conducted. Quarterly data on PCPFO procedure volumes from 1 April 2006 to 31 March 2012 was extracted from the English patient-level Hospital Episode Statistics (HES) database managed by the Health and Social Care Information Centre (HSCIC). HES is a routinely collected data set that records all day case or inpatient episodes of care provided to patients admitted to NHS hospitals and NHS funded patients treated in independent sector hospitals in England. A comprehensive synopsis of HES data is available at http://www.hscic.gov.uk/hes. All index cases were identified using the relevant (K16.5: Percutaneous transluminal closure of patent foramen ovale with prosthesis) Office of Population Census and Surveys (OPCS) procedure code V.4.3.^13^ To identify cases of PCPFO for secondary prevention of cryptogenic stroke/TIA, we excluded patients aged <18 years old and >60 years old as these age groups were excluded from the RCTs^5–7^ and are more likely to have PCPFO due to indications other than secondary prevention of stroke/TIA. In order to estimate procedure rate trends after March 2012, we used the publicly available data provided by the HSCIC^14^ to calculate the average quarterly number of PCPFO procedures per year from 1 April 2006 to 31 March 2013. As these data are aggregated by HSCIC, we were not able to exclude patients based on age in this analysis. We calculated percentage change annualised (PCA) to summarise the diffusion of PCPFO procedures in England over time.^15^ We used Joinpoint trend analysis software to estimate the quarterly percentage change (QPC) in procedure counts and test for changes in that trend during the study period.^16^ The software fits a piecewise regression using weighted least squares with Poisson variance and a grid search method to identify between 0 and 3 joinpoints.

## Results

### Study sample

From the individual patient data, we identified a total of 4388 PCPCO procedures between 1 April 2006 and 31 March 2012. A total of 587 patients aged <18 years old and >60 years were excluded, resulting in a final study cohort of 3801 patients. PCPFO was used in 1897 (49.9%) men and 1903 (50.1%) women with a mean age of 42.6 (SD=10.2) years (table 1). A further 688 patients of all ages had PCPFO between 1 April 2012 and 31 March 2013.

**Table 1:** Patients demographics (n=3801), 2006–2012

Demographics	Number (%)
Sex	
Male	1897 (49.91)
Female	1903 (50.07)
Age group	
18–25	255 (6.71)
26–35	681 (17.92)
36–45	1257 (33.07)
46–60	1609 (42.33)
Race	
White	2979 (78.37)
Others	244 (6.42)
Missing	578 (15.21)

### Trends in the adoption of PCPFO

**Figure 1:**
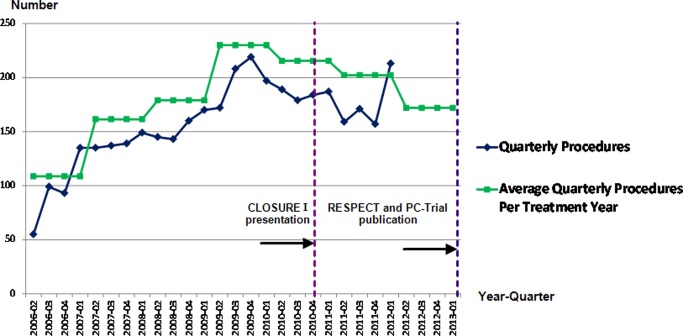
Trends in the adoption of percutaneous closure of patent foramen ovale (PCPFO), 2006–2013*. *Average quarterly procedures (Q2, 2006 to Q1, 2013) based on aggregate data on PCPFO procedures in patients of all ages; quarterly procedures (Q2, 2006 to Q1, 2012) based on individual patient data on PCPFO procedures in patients aged 18–60 years only.

Between the start of the study period (Q2, 2006) and the peak in procedure volume (Q4, 2009), 2163 PCPFO procedures were performed, with a rapidly increasing PCA of 48.4% (figure 1). In the remainder of the study period (Q1, 2010 to Q1, 2012), 1638 PCPFO procedures were performed. Between the peak (Q4, 2009) and the nadir (Q4, 2011) after dissemination of initial trial results, the PCA declined (PCA=−15.3%). Analysis of aggregate data (without age exclusions) suggests that procedure rates fell further during 2012/2013 (figure 1). Data after March 2012 is an overestimate as it is based on national data which does not restrict PCPFO to those ages (18–60) thought most likely to be treated for cryptogenic stroke/TIA. Joinpoint regression identified two inflection points in procedure count trends. The initial rapid increase in procedure counts (QPC=23.9; 95% CI 0.8 to 54.6) attenuated after Q1 2007 until Q4, 2009 (QPC=4.2; 95% CI 1.8 to 6.6) after which procedure rates declined (QPC=−1.2; 95% CI 3.6 to 1.2).

The decline in procedure rates began before the initial conference presentation of results from the first RCT, CLOSURE 1, in November 2010.^17^ CLOSURE 1 was published in March 2012, followed by RESPECT and PC-Trial in March 2013. These trials randomised more than 2300 patients; the only RCT (PC-Trial) that recruited from European centres, included 71 patients recruited from four English hospitals (Professor Bernhard Meier, 2014).

## Discussion

After the publication of NICE guidance on PCPFO in January 2005, the adoption of PCPFO rose rapidly despite the lack of any RCT evidence of efficacy. More than 2000 procedures were performed before RCT results became available in November 2010. Despite NICE's recommendation that PCPFO be used in audit and with further research, only 71 patients from 4 English hospitals were randomised in the first 3 RCTs of PCPFO. The procedure rate peaked before the presentation of the equivocal results from the first RCT.^17^ Since then, the adoption of PCPFO had been falling until the end of the study period in March 2013. It remains unclear whether the current number of PCPFO procedures is appropriate, however, there is clear evidence that NICE guidance published in 2005 was associated with premature adoption of PCPFO before the efficacy was established and without stimulating sufficient engagement with RCTs.

Premature adoption can be defined as widespread technology uptake without adequate evidence of relative advantage, such as safety and cost-effectiveness. Adapted from the Rogers’ S-shaped model of diffusion,^18^ the Balliol Collaboration suggest the peak rate of surgical innovation diffusion may occur once the first 10–20% of surgeons embrace the innovation and that the opportunity for formal assessment might then be lost.^19^ In this context, it is notable that enrolment to the three published PCPFO RCTs and other ongoing trials has lagged considerably, prompting a call for greater engagement among clinicians.^20^ Reasons for slow recruitment include ‘off-label’ use of PCPFO in patients considered too frail or with too many comorbidities for trial enrolment^21^ as well as general clinician and patient barriers to RCT recruitment.^22^

It is likely that some patients treated with PCPFO in England were included in prospective registries and observational studies; therefore contributed to knowledge about adverse events and outcomes, although registries and observational studies are not well-suited for establishing treatment effectiveness. In the UK, there has been an ongoing effort to collect PCPFO complications and clinical outcome data through the establishment of the Central Cardiac Audit Database^12^ and recently the UK Percutaneous Patent Foramen Ovale Closure in adults (PFOC) registry.^23^ However, we are not aware of any peer-reviewed publications on PCPFO from these data sources. To the best of our knowledge, there had only been limited number of registries^24^ ^25^ and observational studies^26–30^ published from England, with a total of 899 patients of 3801 patients in our study cohort, highlighting the inefficiency in the enrolment of NHS patients into research studies.

In the case of PCPFO and many other medical innovations, premature adoption is encouraged by promising results from observational studies.^31^ For example, a meta-analysis study demonstrated that the HR of recurrent stroke in the three RCTs was 0.62 (95% CI 0.34 to 1.11) compared to 0.23 (95% CI 0.11 to 0.49) in observational studies of PCPFO,^9^ suggesting bias and overestimation of the efficacy of PCPFO in early cohort studies.^32^ ^33^ Other case studies of potential premature adoption of cardiac interventional procedures include transcatheter aortic valve implantation (TAVI),^34^ coronary angiography and revascularisation.^35^

Our study has several limitations. First, we are unable to estimate the proportion of the patients who had PCPFO for the secondary prevention of ischaemic stroke/TIA because of the suboptimal coding of previous diagnoses in the HES database.^36^ Nevertheless, we believe that, after age criteria are applied, PCPFO for the secondary prevention of stroke/TIA constitutes the large majority of cases within our study. A large cohort study on 207 PCPFO cases showed that secondary prevention of cryptogenic stroke was the primary indication for PCPFO (93%).^37^ Second, we are unable to show that there is a temporal increase of PCPFO adoption with NICE guidance implementation in 2005 due to unavailability of PCPFO OPCS procedure code before April 2006. However, we believe that NICE guidance had played a major role because it has been the intention of NICE interventional programme guidance to encourage and foster medical innovation.^38^ Third, we do not know to what extent improved coding accuracy, for example due to the introduction of payment by results hospital reimbursement,^39^ might have contributed to the increases in PCPFO procedure rates reported by hospitals. Nevertheless, we believe that this effect is limited as this reimbursement system was first introduced in 2002.^39^ Finally, we cannot be certain whether the slow decline in procedure rates between 2011 and 2013 solely reflects caution among clinicians after the first equivocal RCT results or restrictions on funding introduced by healthcare commissioners in times of economic austerity. Although graphically the decrease of PCPFO adoption does not seem to be temporally related to the presentation of CLOSURE I results, we believe that the presentation of the CLOSURE I results had at least to some degree contributed to the decline, alongside with these limitations due to the multifactorial causes of PCPFO adoption.

### Implications and conclusion

Premature adoption of unproven medical innovation is a form of low value care as unproven medical device with uncertain risk-to-benefit ratio^40^ offers little or no net clinical benefits in relative to the alternative treatments.^41^ It is an avoidable waste of healthcare resources and worse still could be harmful to patients.^42^ Recent scandals^43^ have prompted a change in the EU legislation on medical devices whereby high quality evidence is required for medium-risk and high-risk procedures before market approval.^44^ Clinicians must be mindful of embracing nascent medical technology until robust unbiased evidence on the safety and efficacy of new medical technology is established. We recommend the IDEAL framework in the evaluation of new medical innovations.^45^ Evidence generation should follow the hierarchy whereby the beginning of a RCT should mark the end of reliance from observational studies to provide evidence. Studies higher on the hierarchy of evidence, such as RCTs, should be given priority to avoid competition for patient recruitment. Clinicians understandably face challenges in RCT recruitment; support from policymakers and healthcare organisations are essential to overcome these barriers.^22^ In cases where RCTs are not possible due to rare events like in this case, observational studies on postmarketing surveillance of the medical devices are important to capture the outcome data to determine if the benefits of the medical device outweigh the risks.^46^

In conclusion, PCPFO was rapidly adopted after the publication of NICE guidance in 2005 despite the absence of RCT evidence of efficacy. Several thousand patients had the procedure in England before meta-analyses of three RCTs provided statistically weak evidence on the efficacy of PCPFO to reduce recurrent stroke/TIA.^47^ A meta-analysis study which includes the non-randomised studies demonstrated that there is an increasing net clinical benefits of PCPFO over time in terms of stroke/TIA recurrence and bleeding risks compared to the anticoagulant/antiplatelet therapies.^48^ Consequently, a personalised approach where the age of patient, comorbidities, interaction with other medications should be considered when deciding if PCPFO or medical therapy is suitable for a patient. NICE recommendations contributed to premature adoption of PCPFO and failed to encourage the timely generation of RCT evidence. Further studies are required to investigate the factors leading to premature adoption of new medical innovation and how to prevent this phenomenon.
